# The NFAT3/RERG Complex in Luminal Breast Cancers Is Required to Inhibit Cell Invasion and May Be Correlated With an Absence of Axillary Lymph Nodes Colonization

**DOI:** 10.3389/fonc.2022.804868

**Published:** 2022-06-30

**Authors:** Lucie Coillard, Frédéric Guaddachi, Maëlle Ralu, Eva Brabencova, Christian Garbar, Armand Bensussan, Morgane Le Bras, Jacqueline Lehmann-Che, Sébastien Jauliac

**Affiliations:** ^1^ Université de Paris, Research Saint Louis Institute (IRSL), Institut National de la Santé et de la Recherche Médicale, Human Immunology Pathophysiology Immunotherapy (INSERM HIPI) U976, Paris, France; ^2^ Department of Biopathology, Centre Régional de Lutte Contre le Cancer, Institut Godinot, Reims, France; ^3^ Molecular Oncology Unit, Assistance Publique-Hôpitaux de Paris (AP-HP), Hôpital Saint Louis, Paris, France

**Keywords:** breast cancer, NFAT3/c4, RERG, invasion, lymph nodes metastasis

## Abstract

Luminal breast cancers represent 70% of newly diagnosed breast cancers per annum and have a relatively good prognosis compared with triple-negative breast cancers. Luminal tumors that are responsive to hormonal therapy are particularly associated with a favorable prognosis. Nonetheless, the absolute number of metastatic relapses in luminal cancers is larger than in triple-negative breast cancers. A better understanding of the biology of luminal cancers, control of metastases formation, and identification of predictive markers of their evolution are therefore still necessary. In this context, we previously disclosed the key role of NFAT3 in regulating luminal breast cancer invasion. We have now identified a specific inhibitory region, in the C-terminal part of NFAT3, required for the inhibition of invasion of the human luminal breast cancer cell line T-47D. Indeed, we showed that this 85 amino acid C-terminal region acts as a dominant negative form of NFAT3 and that its overexpression in the T-47D cell line led to increased cell invasion. Mechanistically, we have revealed that this region of NFAT3 interacts with the small Ras GTPase RERG (RAS like estrogen regulated growth inhibitor) and shown that RERG expression is required for NFAT3 to impede T-47D cell invasion. We have validated the association of NFAT3 with RERG in human luminal breast cancer tissues. We have shown an increase of the quantity of the NFAT3/RERG complexes in patients without axillary lymph node colonization and therefore proposed that the detection of this complex may be a non-invasive marker of axillary lymph node colonization.

## Introduction

Breast cancer is still a major cause of cancer-related death in women. This morbidity often relies on the potency of the breast tumors to develop distant metastases. One of the characteristics of breast cancers is their high molecular heterogeneity (1) where classically, the triple-negative subtype has a worse prognosis compared to the luminal one. This poor prognosis is directly related to the high rate of metastases formation in triple-negative tumors. However, even if the luminal subtype has a relatively good prognosis, it is the most frequently diagnosed and represents a proportion of 70% of all identified breast cancers. Because of this overrepresentation, the absolute number of metastatic relapses in luminal cancers is larger than in triple-negative breast cancers. Indeed, as recently shown by Maaren et al. (2), in ten-year recurrences, the number of patients with metastases represents 10% of the luminal subtype whereas this number falls to 4% for the other subtypes. There is therefore a real need for predictive tools to distinguish between luminal tumors that will be more susceptible to metastasize and those that will not metastasize. These predictive tools associated with other clinicopathological parameters would be valuable assets for decisions concerning optimal treatment. Moreover, elucidating the mechanisms of action of pro- or anti-metastatic factors expressed in different breast cancer subtypes would be a useful approach to potentially identify new targetable pro- or anti-oncogenic pathways.

In this context, our group identified that the isotypes of the NFAT transcription factors family are differentially expressed between breast cancer subtypes and have opposite effects on metastatic dissemination. Indeed, NFAT1 (NFATc2) exerts a pro-invasive function and is mainly expressed in the triple-negative subtype, whereas NFAT3 (NFATc4) has anti-invasive properties, limiting the aggressiveness of primary NFAT3-expressing luminal breast cancer cells (3–6). Additionally, we recently showed that NFAT3-expressing cells can produce anti-tumoral and anti-metastatic NFAT3-directed extracellular vesicles (7). Considering the high amino acid sequence homology between NFAT1 and NFAT3, it is puzzling that these two isotypes have clearly opposed effects on tumor growth and metastasis formation. Unraveling the origins and molecular mechanisms of their antagonistic functions in breast cancer could be a key contribution to better understand their respective roles in tumor progression. The most obvious reason for these specific functions may rely upon their abilities to interact with specific protein partners. Indeed, many NFAT protein partners with a direct role on isotype function have been reported (8–11). Apart from their specific functions in breast cancer, the differential expression of NFAT1 in the triple-negative subtype and of NFAT3 in luminal cancers opens the possibility that these factors may be potential prognostic markers with NFAT3 protein expression for tumors that will not generate distant metastasis.

Among these putative prognostic markers, RERG (12), a growth-inhibitory gene highly expressed in luminal breast cancer, was correlated with the estrogen-regulated longest survival of luminal breast cancer patients (13) without metastases.

Here, we report that NFAT3 specifically interacts, at least *via* its last 85 C-terminal amino acids, with RERG in the luminal breast cancer cell line T-47D. This interaction is functional and is required for NFAT3 to inhibit the invasion of this breast cancer luminal cell line. We confirmed the presence of this association in luminal breast cancer tissues from patients. Finally, we report that tumors with axillary lymph nodes (ALN) colonization (N+) had fewer detectable NFAT3/RERG complexes compared to those without ALN infiltration (N0). Together these results provide new insights in the anti-tumoral effects of NFAT3 and its association with RERG and also highlight the potential value of the detection of the NFAT3/RERG association in luminal breast tumor patients as an indicator of the ALN status.

## Materials and Methods

### Cell Lines

T-47D and NIH3T3 cell lines were obtained from the ATCC. All cell lines were validated as mycoplasma negatives by PCR and grown in an Roswell Park Memorial Institute medium (RPMI) 1640 medium (T-47D) plus 10% fetal bovine serum (PAN BIOTECH) or in high-glucose (4.5 g/L) Dulbecco’s Modified Eagle Medium (DMEM) (NIH3T3) plus 10% newborn calf serum (PAN BIOTECH) and were maintained in a 5% CO_2_ incubator at 37°C.

### Antibodies and Reagents

Antibodies used in the study were α-HA (Roche Cat# 11867423001, RRID: AB_390918), α-NFAT3 [(Sigma-Aldrich Cat# HPA031641, RRID: AB_10600826), for Proximity Ligation Assay (PLA)], α-RERG (Sigma-Aldrich Cat# SAB1403408, RRID: AB_10737054, for PLA), α-pan cytokeratin (Sigma-Aldrich Cat# F3418, RRID: AB_259536); α-actin (Thermo Fisher Scientific Cat# MA5-15739, RRID: AB_10979409), α-NFAT3 (Thermo Fisher Scientific Cat# PA1-021, RRID: AB_2267268, for Western blot); α-RERG (Proteintech Cat# 10687-1-AP, RRID: AB_2253772, for Western blot); α-HA (Abcam Cat# ab18181, RRID: AB_444303, For PLA). ReadyTector α-Mouse-HorseRadish Peroxidase (HRP) #720 500 and α-Rabbit-HRP #730 500 were from Candor. Lipofectamine 2000 #11668019 from Thermofisher Scientific; Dharmafect 1 #T-2001-03 and siNFAT3 #D-009584-07, siRERG #L-0082004; siCtl #D-001810-10 and # D-001210-01 were from Horizon.

### Plasmids and Generation of NFAT3 Expression Constructs

All the deletion mutants generated by PCR were cloned into the pcDNA3.1 (+) vector with the Hemagglutinin (HA) epitope for NFAT3, NFAT3-85C, ΔNFAT3, ΔNFAT3-85c, and NFAT3-Cter and verified by sequencing. The plasmid pCS2-(n)-β-galactosidase has been described previously (3). The pTRIP plasmid was provided to us by P. Charneaux (Institut Pasteur, Paris, France), the ReMTH plasmids by Sonyang Z. (Sun Yat-sen University, Canton, China) the Venus construct by A. Miyawaki (Riken, Tokyo, Japan), and RERG expression vector by C.M. Perou (University of North Carolina, Chapel Hill, North Carolina, USA).

### Western Blot

Whole cell lysates were obtained by boiling 0.5.10^6^ cells in the reducing Laemmli sample buffer. The lysates were resolved by SDS-PAGE and probed with α-NFAT3 #PA1-021,1:1,000 diluted in ReadyTector α-Rabbit-HRP; α-actin, 1:2,500 in ReadyTector α-Mouse-HRP; α-RERG, 1:1,000 in ReadyTector α-Rabbit-HRP. Revelation with α-HA was followed by incubation with Goat α-Rat IgG secondary antibody at room temperature. All primary incubations were performed overnight at 4°C.

### Retrovirus-Based Complementation Assay

The RePCA screen was carried out as described previously (14) with the T-47D cell line infected with the lentivirus pTRIP stably expressing the fusion of the N-terminal part of Venus fused to the C-terminal part of NFAT3 (VNN-N3Cter) infected with the ReMTH-VC (retrovirus-based molecular) retrovirus containing the C-terminal part of Venus upstream of a splice donor site in the 3 open reading frames. The infected cells were selected by treatment with puromycin, and the fluorescent ones were sorted individually by Fluorescence Activated Cell Sorter (FACS). Total RNA from sorted cells was extracted with TRIzol (Ambion, Austin, Texas, USA). Reverse transcription was performed with the Superscript II kit (Life Technologies, Eugene, Oregon, USA) and random primers containing the T7 tag (5′-TAATACGACTCACTATAGGGNNNNNNN-3′). The complementary DNA (cDNA) was amplified by PCR with a primer hybridizing to the C-terminal part of Venus (5’-CCACTACCTGAGCTACCAGTCC-3’) and a T7 primer (5’-GCGCTAATACGACTCACTATAGGG-3’). The PCR products were gel purified and sequenced and identified by Basic Local Alignment Search Tool (BLAST).

### Invasion Assays, Immunofluorescence, and Proximity Ligation Assay

Invasion assays were performed essentially as previously described (5). For immunofluorescence and PLA, cells were grown on coverslips in 24-well plates and transfected with the relevant siRNA or plasmids. Approximately 48 h after transfection, cells were labeled for PLA. Slides were fixed in 4% paraformaldehyde for 15 min, washed 3 times in PBS 100 mM glycine, and permeabilized for 10 min in PBS Triton 0.2%. Saturation for non-specific binding was carried out in the blocking buffer [PBS, 5% Bovine Albumin Serum (BSA)] for 45 min at room temperature. Then, the slides were incubated with α-NFAT3 (1:200) and α-RERG, (1:50) or α-HA (# ab18181, 1:500) in the blocking buffer overnight at 4°C. The following day, slides were washed and incubated with the Plus and Minus probes of the Duolink kit (#DUO92101, Sigma-Aldrich) diluted in the blocking buffer. The kit was used as indicated by the manufacturer. The same protocol was followed for the frozen tissues following addition, after the last washes of the Duolink reaction, of an α-pan cytokeratin coupled to Fluorescein IsoThioCyanate (FITC) (1:100) in the blocking buffer overnight at 4°C. The α-pan cytokeratin allowed us to identify the breast epithelial carcinoma cells. The following day, slides were washed and mounted with a Fluoromount G medium (SouthernBiotech, Homewood, Alabama, USA).

### Microscope Image Acquisition

*Cell lines:* Fluorescence images were captured using a Microscope Axio Imager.D2 equipped with a Plan Apochromat 63X N.A.1.4 oil immersion objective: room temperature with an AxioCamMR3 and the Axiovision acquisition software.

*Tissues:* Fluorescence images were acquired by confocal microscopy on a Zeiss LSM 800 confocal laser microscope (Zeiss) using a Plan Apochromat 63X N.A.1.4 oil immersion objective using the ZEN software (Zeiss). For both cell lines and tissues, both the control and the sample images were acquired using the same settings. The settings of acquisition were optimized to avoid the saturation of signals. PLA dots were counted using the particle analysis function of ImageJ for both cell lines and tissues. For the cell lines, the number of nuclei were counted manually and the PLA index was obtained by dividing the number of PLA dots by the number of the nuclei. For tissues, in order to minimize bias, as the coalescence of the nuclear signal prevented accurate counting of the nuclei in cytokeratin-positive cells, we manually measured with ImageJ the DAPI-positive surface of the cytokeratin-positive cells. Consequently, the index of PLA for the tissues was obtained by dividing the number of PLA dots by the surface of the nuclei of cytokeratin-positive cells.

### Human Luminal Breast Cancer Tissues

All the tissue ER+ samples (N+ and N0) had been flash-frozen between 2011 to 2014 and were stored at -80°C. Frozen tissue specimens were collected from the Biobank of the biopathology department of Godinot Institute, Reims, France, for 21 luminal breast cancer patients with ALN colonization [n=10: 2014 (2), 2013 (4), 2012 (1), 2011 (3)] or without [n=11: 2014 (2), 2013 (4), 2012 (3), 2011 (2)]. We included all available ER+ breast primary tumor tissues without selection. All patients gave informed written consent. The research was approved by the Ethics Committee of Saint Louis Hospital and the Godinot Institute.

### Statistical Analysis

The error bars in the graphical data represent the means ± SEM. When relevant, p-values were obtained by an unpaired, two-tailed Student’s t-test using GraphPad Prism software (GraphPad Software Inc., La Jolla, CA, USA).

## Results

### The Last 85 C-Terminal Amino Acids of NFAT3 Are Required to Inhibit Cell Invasion

**Figure 1:**
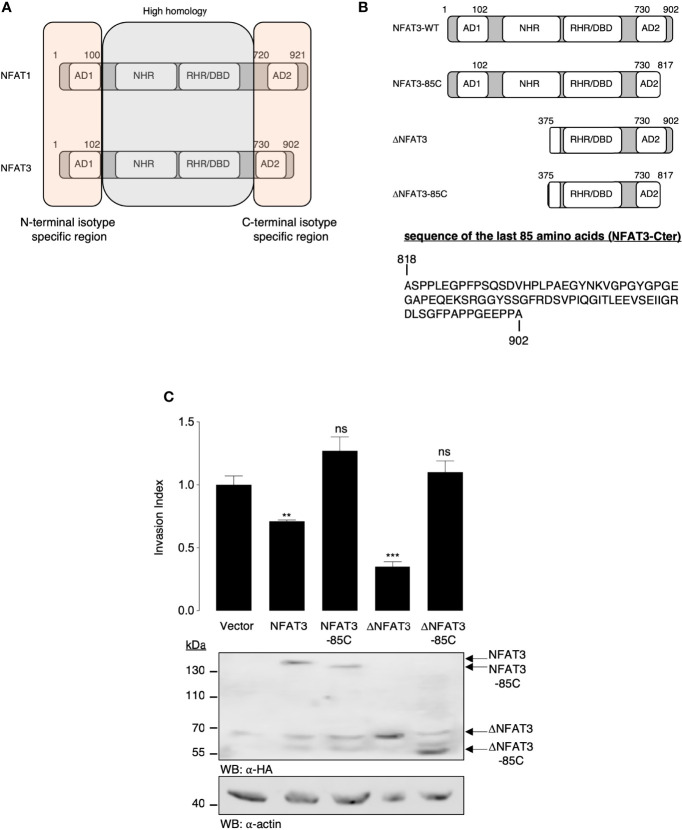
The last 85 C-terminal amino acids of NFAT3 are required for the inhibition of cell invasion. **(A)** Schematic representation of the homologous and isotype-specific regions of human NFAT1 and NFAT3 with the activation domains (ADs), the NFAT homology domain (NHR), the Rel homology domain (RHR), and the DNA-binding domain (DBD). **(B)** Schematic representation of the NFAT3, NFAT3-85C, ΔNFAT3, and ΔNFAT3-85C constructs used in the study and the sequence of the last 85 amino acids of NFAT3 (NFAT3-Cter). **(C)** T-47D cells were transiently transfected with either a vector encoding the HA epitope fused to NFAT3, NFAT3-85C, ΔNFAT3, or ΔNFAT3-85C or with the empty vector (Vector). The following day, transfected cells were subjected to an *in vitro* invasion assay for 24 h. All data are shown as the mean of two independent experiments ± SEM (n=2 biological replicates; 3 technical replicates for each experiment, **p<0.005, ***p< 0.0001, ns, non specific relative to the vector-transfected cells). Whole cell lysates were revealed by α-HA and normalized by revelation with an α-actin.

NFAT1 and NFAT3 amino acid sequences are highly homologous in the central region containing the NHR and the RHR domains, while they show an isotype specificity in the first 100 N-terminal and the last 200 C-terminal amino acids (**Figure 1A**). We thus hypothesized that the specific function of the pro-invasive (NFAT1) (3, 5, 6) or anti-invasive (NFAT3) (4, 7) capacity could be linked to these unique regions. We chose to specifically focus on the NFAT3 anti-invasive capacity as we have already shown that deleting its first 374 amino acids to generate the ΔNFAT3 deletion mutant did not prevent its anti-invasive function (4). Accordingly, we suggested that the anti-invasive activity of NFAT3 might rely on its C-terminal region. To validate this hypothesis, we generated C-terminal truncated NFAT3 and ΔNFAT3 mutants (**Figure 1B** **)** and determined that the last 85 C-terminal amino acids were required for both NFAT3- and ΔNFAT3-inhibited cell invasion as shown in **Figure 1C**. We did not identify any specific domains in the NFAT3-Cter. The last 85 C-terminal amino acids, lacking specific functional domains, were referred to as NFAT3-Cter for the rest of the study.

### Overexpression of NFAT3-Cter Is Sufficient to Increase Breast Cancer Cell Invasion

**Figure 2:**
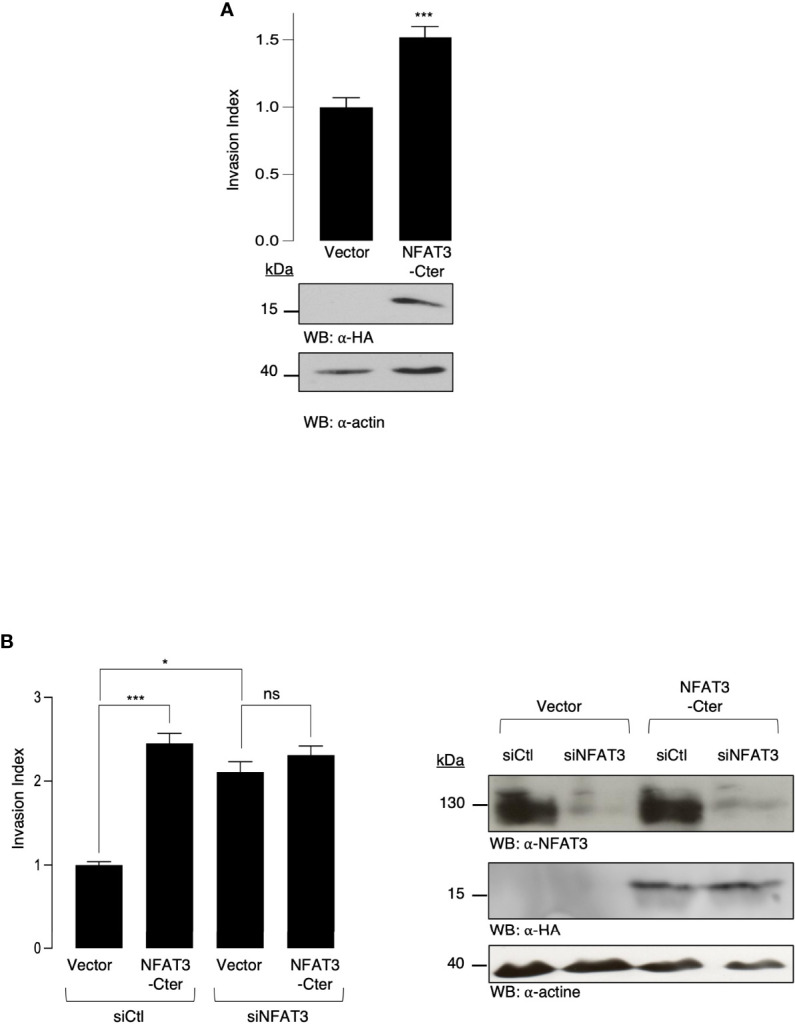
NFAT3-Cter acts as a dominant negative form of endogenous NFAT3. **(A)** T-47D cells were transiently transfected with either a vector encoding the HA epitope fused to NFAT3-Cter or with the empty vector (Vector). The following day, transfected cells were subjected to an *in vitro* invasion assay for 24 h. All data are shown as the mean of three independent experiments ± SEM (n=3 biological replicates; 3 technical replicates for each experiment; *p<0.005). Whole cell lysates were revealed by α-HA and normalized by revelation with α-actin. **(B)** T-47D cells were transiently cotransfected with either an siRNA control (siCtl) or an siRNA targeting the endogenous NFAT3 (siNFAT3) in combination with either a vector encoding the HA epitope fused to NFAT3-Cter or with the empty vector (Vector). Approximately 48 h later, transfected cells were subjected to an *in vitro* invasion assay for 24 h. The mean of three independent experiments ± SEM is shown (n=3 biological replicates; 3 technical replicates for each experiment; *p<0,05, ***p<0,001, ns, non-specific). Whole cell lysates were revealed by α-HA and α-NFAT3 and normalized by revelation with α-actin.

To elucidate the mechanisms underlying the requirement for the inhibitory NFAT3-Cter region (NFAT3-Cter), we overexpressed this region in T-47D cells and evaluated their invasive capacity. The overexpression of the NFAT3-Cter did not inhibit breast cancer cell invasion but significantly increased it compared to the cells transfected with the empty vector (**Figure 2A**). We then hypothesized that the overexpressed NFAT3-Cter region could have a dominant negative effect on the endogenous NFAT3. Indeed, the results presented in **Figure 2B** indicate that the depletion of the endogenous NFAT3 protein by a specific siRNA prevented the increased invasion induced by the NFAT3-Cter region. Altogether, these results suggested that the pro-invasive function of the NFAT3-Cter region behaved as a dominant negative form of NFAT3.

### Identification of RERG as an NFAT3-Cter Interacting Protein

**Figure 3:**
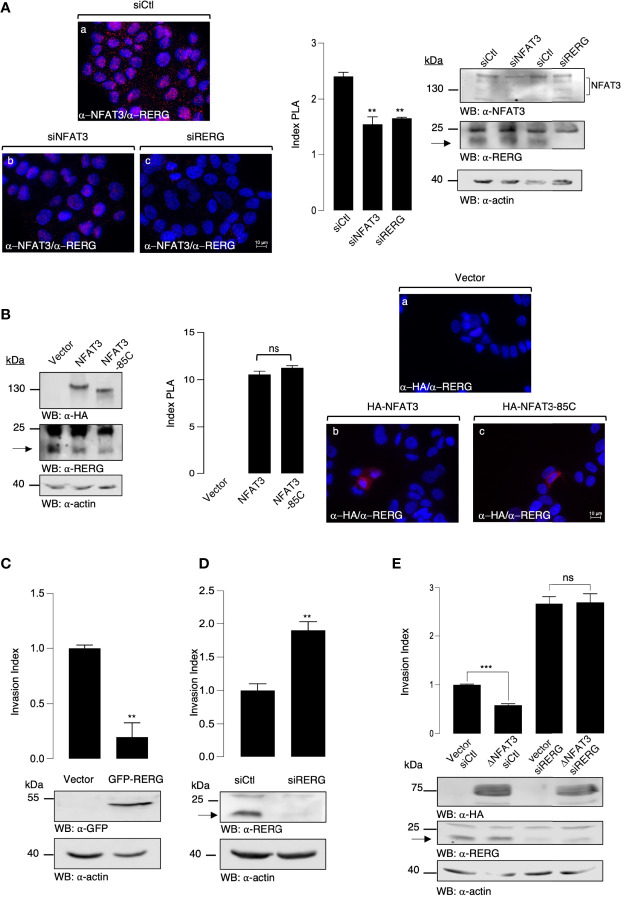
NFAT3 is associated with RERG in T-47D cells to suppress breast cancer cell invasion. **(A)** T-47D cells were grown on coverslips and transfected with either an siRNA control (siCtl), a siRNA targeting the endogenous NFAT3 (siNFAT3), or the endogenous RERG (siRERG). Approximately 48 h after transfection PLA assays were performed on the transfected cells with the anti-NFAT3 and the anti-RERG (α-NFAT3/α-RERG) (a-c). Index of PLA was obtained by analyzing ten random fields for each condition, all data are shown as the mean of three independent experiments ± SEM (n=3 biological replicates; 3 technical replicates for each experiment; **p<0.005, ***p<0,001, ns, nonspecific). Whole cell lysates were revealed by α-NFAT3 and α-RERG and normalized by comparison with an α-actin. **(B)** T-47D cells were grown on coverslips and transfected with either the empty vector or a vector encoding the HA epitope fused to either NFAT3 or NFAT3-85C. Approximately 24 h after transfection, PLA assays were performed on the transfected cells with the α-HA and α-RERG (a-c). Index of PLA was obtained by analyzing fifteen random fields for each condition; all data are shown as the mean of three independent experiments ± SEM (n=3 biological replicates; 10 random fields for each condition; ns = non-specific). Whole cell lysates were revealed by α-HA and α-RERG and normalized by revelation with an α-actin. **(C)** T-47D cells were transiently transfected with either a vector encoding the GFP protein fused to RERG or with the empty vector (Vector). The following day, transfected cells were subjected to an *in vitro* invasion assay for 24 h. Data are shown as the mean of three independent experiments ± SEM (n=3 biological replicates; 3 technical replicates for each experiment; ***p<0.001). Whole cell lysates were revealed by α-GFP and normalized by revelation with α-actin. **(D)** T-47D cells were transiently transfected with either an siRNA control (siCtl) or an siRNA targeting the endogenous RERG (siRERG). Approximately 48 h after the transfected cells were subjected to an *in vitro* invasion assay for 24 h, all data are shown as the mean of three independent experiments ± SEM (n=3 biological replicates; 3 technical replicates for each experiment; ***p<0.001, ns, non-specific). Whole cell lysates were revealed by α-RERG and normalized by revelation with α-actin. **(E)** T-47D cells were transiently co-transfected with either an siRNA control (siCtl) or an siRNA targeting the endogenous RERG (siRERG) in combination with either a vector encoding the HA epitope fused to ΔNFAT3 or with the empty vector (Vector). Approximately 48 h later, the transfected cells were subjected to an *in vitro* invasion assay for 24 h. Data are shown as the mean of three independent experiments ± SEM (n=3 biological replicates; 3 technical replicates for each experiment; ***p<0.001, ns, non-specific). Whole cell lysates were revealed by α-HA and α-RERG and normalized by revelation with α-actin.

Dominant negative constructs generally act on endogenous proteins by modulating their association with other factors. Therefore, we hypothesized that the overexpression of NFAT3-Cter reduced available inhibitory co-factors, required to impede cell invasion, and are usually associated with the endogenous NFAT3 C-terminal region. To identify these cofactors, we performed a retrovirus-based complementation assay (RePCA) screen as described by Ding et al. (14). The bait was the NFAT3-Cter region fused to the N-terminal half of the Green Fluorescent Protein (GFP). T-47D cells were stably infected by a lentivirus containing this bait. The resulting stable clones were then infected with a retrovirus carrying a vector containing the half C-terminal of the GFP cloned upstream of a splice donor site (prey). The resulting green cells indicated that the NFAT3-Cter region interacted with the prey. GFP-positive cells were sorted, and genomic DNA was extracted and sequenced. BLAST analysis was performed to identify interacting proteins. Among the different proteins, we identified a fusion of the GFP C-terminal half with the second exon of RERG (data not shown). We chose to focus on the association of RERG with NFAT3 because previous studies have associated RERG expression with better survival in luminal breast cancer patients (13) and revealed its capacity to inhibit cell invasion (15). To confirm the potential association of the endogenous NFAT3 and RERG in T-47D cells, we performed PLA assays after the downregulation of either NFAT3 or RERG, using specific siRNAs. Both siRNAs against NFAT3 and RERG were competent to reduce protein expression compared with a control siRNA (**Figure 3A**, left panel). In cells transfected with a control siRNA, we observed red dots, indicating the association of NFAT3 with RERG (**Figure 3A**, (a) and index PLA). This signal was reduced in cells transfected with either the NFAT3 or the RERG siRNA (**Figure 3A**, (b, c) and index PLA), underlining the specificity of this association. Moreover, we showed that the deletion of the NFAT3-Cter region was insufficient to prevent NFAT3 association with endogenous RERG (**Figure 3B**, (c) and index PLA). These results indicate that NFAT3 and RERG are associated in T-47D cells, with NFAT3-Cter being dispensable for this association. This suggests that other regions of NFAT3 may participate with the NFAT3-Cter region in this association.

### NFAT3 Requires RERG to Suppress Breast Cancer Cell Invasion

As previously reported in other models (16, 17), we confirmed that the overexpression of RERG inhibits cell invasion (**Figure 3C**) and that the siRNA-mediated downregulation of RERG enhanced the invasion **(** **Figure 3D** **)**. To determine the functional role of RERG binding to the last 85 amino acids of NFAT3, we tested whether NFAT3 was dependent on RERG to inhibit cell invasion. To do so, we cotransfected T-47D cells with either a control siRNA (siCtl) or an RERG siRNA (siRERG) along with either a control empty vector (Vector) or the active deletion mutant of NFAT3 (ΔNFAT3). The results presented in **Figure 3E** show that ΔNFAT3 was no longer able to inhibit breast cancer cell invasion when endogenous RERG was downregulated. These results, alongside those obtained from the PLA experiments, demonstrate that the presence of RERG and its association with NFAT3 *via* the Cter region is mandatory for NFAT3 to impede breast cancer cell invasion. This revealed the functional association between NFAT3 and RERG in the T-47D luminal breast cancer cell line.

### NFAT3/RERG Interaction Is Increased in Luminal Breast Cancer Tissues and Correlated With the Absence of Axillary Lymph Node Colonization

**Figure 4:**
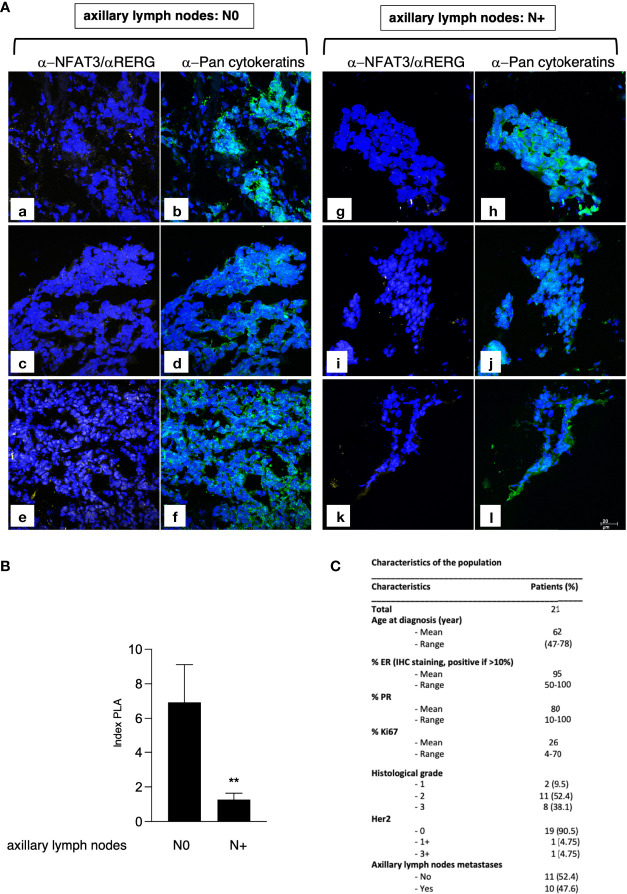
NFAT3/RERG interaction is increased in luminal breast cancer tissues and is correlated with the absence of ALN colonization. **(A)** Frozen luminal breast cancer sections from patients who developed ALN colonization (N+; a-f) or not (N0; g-l) were incubated with α-NFAT3 and α-RERG followed by PLA assays (yellow dots) and labeling with α-pan cytokeratin coupled to FITC. Data from 6 representative patient slides out of 21 are shown. **(B)** Index of PLA was obtained by analyzing 2 random fields for the 21 slides; data are shown as mean ± SEM (**p<0,005). **(C)** Characteristics of the patients used for microscopy.

We further validated the NFAT3 interaction with RERG by PLA assays (**Figure 4A**) in luminal breast cancer tissues (**Figure 4C**) obtained from patients with (10 patients) or without (11 patients) distant ALN metastases at diagnosis. This association was present in almost all the luminal breast cancer tissues tested so far, at different levels, independently of the N0 or N+ status of the patients (data not shown). Remarkably, the number of NFAT3/RERG complexes was statistically increased in tissues from patients with no ALN colonization (N0) compared with patients with ALN colonization (N+) (**Figures 4A, B**). These results suggest that the detection of NFAT3/RERG complex may constitute a new prognostic marker of the absence of ALN colonization.

## Discussion

The recurrence of luminal breast cancer subtypes generally occurs later than the recurrence of triple- negative cancers. This therapeutic situation underlines the urgency of improving both our knowledge of the fundamental mechanisms regulating breast cancer cell invasion/metastases and the discovery of additional prognostic markers. Such progress could eventually enhance the pathological complete response (pCR) by a patient-tailored treatment and individualized follow-up.

In this context, we have previously uncovered the anti-invasive role of NFAT3 in luminal breast cancer (4) and shown that extracellular vesicles (EVs) produced by NFAT3-expressing luminal breast cancer cells were competent to prevent tumor growth and restrain metastases spreading in a murine triple- negative breast cancer model (7). Regarding the expression and function of NFAT isotypes in breast cancer, we have highlighted that NFAT3 and NFAT1 had absolutely opposed effects on breast cancer cell invasion, NFAT3 being anti-invasive and NFAT1 pro-invasive (3–6). These last observations were puzzling since NFAT3 and NFAT1 are highly homologous in terms of amino acid sequence.

In the present study, we aimed to understand the structural requirements for NFAT3 to impede breast cancer cell invasion. Therefore, we focused our attention on the N- and C-terminal sequences of NFAT1 and NFAT3 that had isotype specificity (**Figure 1A**) and identified a specific region of 85 amino acids, in the C-terminal part of NFAT3, required for the inhibition of cell invasion of the human luminal breast cancer cell line T-47D. Numerous studies have shown that the NFAT factors interact with multiple cofactors to modulate their functions (8–11, 18–26). Indeed, because of the dominant negative effect of the last 85 amino acid C-terminal of NFAT3 on the invasive capacity of this luminal breast cancer cell line, we hypothesized co-factor binding to this specific region to inhibit cell invasion and revealed its association with RERG. We confirmed that this NFAT3/RERG complex, *via* the NFAT3-Cter region, was functional and necessary for NFAT3 to impede invasion in the human luminal T-47D breast cancer cell line. Future studies will reveal whether this is also the case in other luminal breast cancer cell lines than T-47D. RERG is a growth-inhibitory gene highly expressed in luminal breast cancer (12) and is associated with the longest survival of luminal breast cancer patients (13) without metastases. Some studies have reported that RERG is an inhibitor of the MAPK/ERK pathway (15), a pathway implicated in breast cancer cell migration (27, 28). However, further investigations are required to study the potential role of the ERK pathway in the anti-invasive effect of NFAT3.

Importantly, we validated the association of NFAT3 with RERG in luminal breast cancer tissues from patients and disclosed that a higher amount of this complex, in the primary tumor, was observed in patients lacking axillary lymph node colonization. Axillary lymph node colonization remains a strong prognostic factor for predicting prognosis in breast cancer patients (29, 30). Thus, our study suggests that detection of NFAT3/RERG complex in the primary tumor could be a new prognostic marker of the absence of ALN colonization. Further studies with a larger cohort of patients will be necessary to definitively confirm the potential use of the NFAT3/RERG association as a valuable non-invasive indicator of the ALN status.

## Data Availability Statement

The original contributions presented in the study are included in the article/**Supplementary Material** Further inquiries can be directed to the corresponding author.

## Ethics Statement

The studies involving human participants were reviewed and approved by Ethics Committee of Saint Louis Hospital, Ethics Committee of the Godinot Institute. The patients/participants provided their written informed consent to participate in this study.

## Author Contributions

SJ and LC contributed to conception and design of the study. SJ, LC, FG, and MR performed the experiments. AB organized the access for the breast cancer tissues. EB and CG provided the breast cancer tissues. SJ wrote the first draft of the manuscript. LC, ML, JL, and MR wrote sections of the manuscript. All authors contributed to manuscript revision, read, and approved the submitted version.

## Funding

Fondation ARC pour la Recherche sur le Cancer: PJA 20131200039 and Groupements des Entreprises Française dans la lutte contre le Cancer (GEFLUC).

## Conflict of Interest

The authors declare that the research was conducted in the absence of any commercial or financial relationships that could be construed as a potential conflict of interest.

## Publisher’s Note

All claims expressed in this article are solely those of the authors and do not necessarily represent those of their affiliated organizations, or those of the publisher, the editors and the reviewers. Any product that may be evaluated in this article, or claim that may be made by its manufacturer, is not guaranteed or endorsed by the publisher.
