# Corrigendum: The NFAT3/RERG complex in luminal breast cancers is required to inhibit cell invasion and may be correlated with an absence of axillary lymph nodes colonization

**DOI:** 10.3389/fonc.2022.1016189

**Published:** 2022-08-30

**Authors:** Lucie Coillard, Frédéric Guaddachi, Maëlle Ralu, Eva Brabencova, Christian Garbar, Armand Bensussan, Morgane Le Bras, Jacqueline Lehmann-Che, Sébastien Jauliac

**Affiliations:** ^1^ Université de Paris, Research Saint Louis Institute (IRSL), Institut National de la Santé et de la Recherche Médicale, Human Immunology Pathophysiology Immunotherapy (INSERM HIPI) U976, Paris, France; ^2^ Department of Biopathology, Centre Régional de Lutte Contre le Cancer, Institut Godinot, Reims, France; ^3^ Molecular Oncology Unit, Assistance Publique-Hôpitaux de Paris (AP-HP), Hôpital Saint Louis, Paris, France

**Keywords:** breast cancer, NFAT3/c4, RERG, invasion, lymph nodes metastasis

In the published article, there was an error in the abstract. NFAT3/REG should be replaced by NFAT3/RERG.

A correction has been made to **Abstract**. The corrected sentence appears below:

“We have shown an increase of the quantity of the NFAT3/RERG complexes in patients without axillary lymph node colonization and therefore proposed that the detection of this complex may be a non-invasive marker of axillary lymph node colonization.”

The authors apologize for this error and state that this does not change the scientific conclusions of the article in any way. The original article has been updated.

## Publisher’s note

All claims expressed in this article are solely those of the authors and do not necessarily represent those of their affiliated organizations, or those of the publisher, the editors and the reviewers. Any product that may be evaluated in this article, or claim that may be made by its manufacturer, is not guaranteed or endorsed by the publisher.

