# Supplementary figures and images for: The NFAT3/RERG Complex in Luminal Breast Cancers Is Required to Inhibit Cell Invasion and May Be Correlated With an Absence of Axillary Lymph Nodes Colonization
Source: Front Oncol. 2022 Jun 30;12:804868. doi: 10.3389/fonc.2022.804868 (PMC9280138; doi:10.3389/fonc.2022.804868)

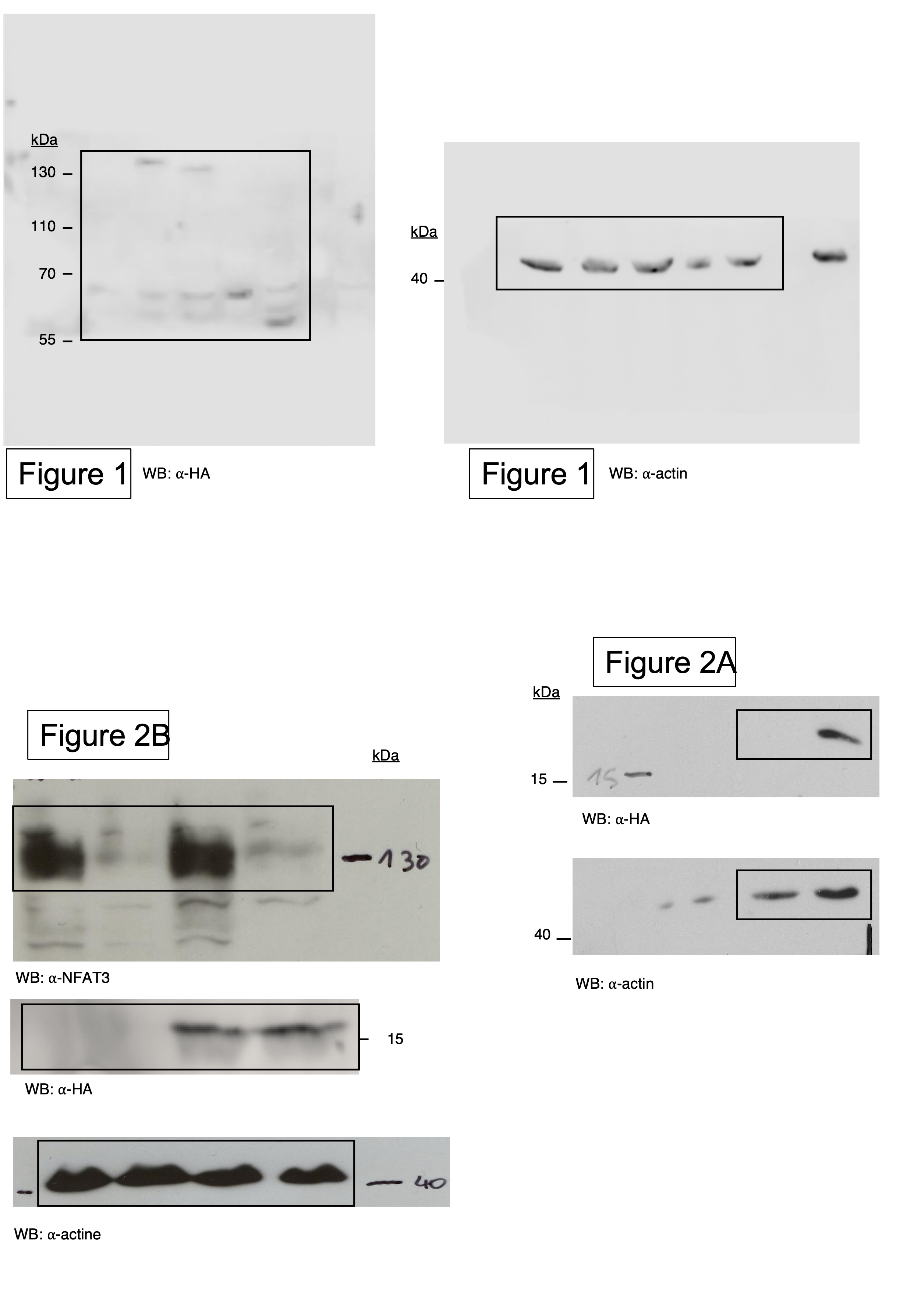

Supplement: Supplementary file 1 [file Image_1.jpeg]

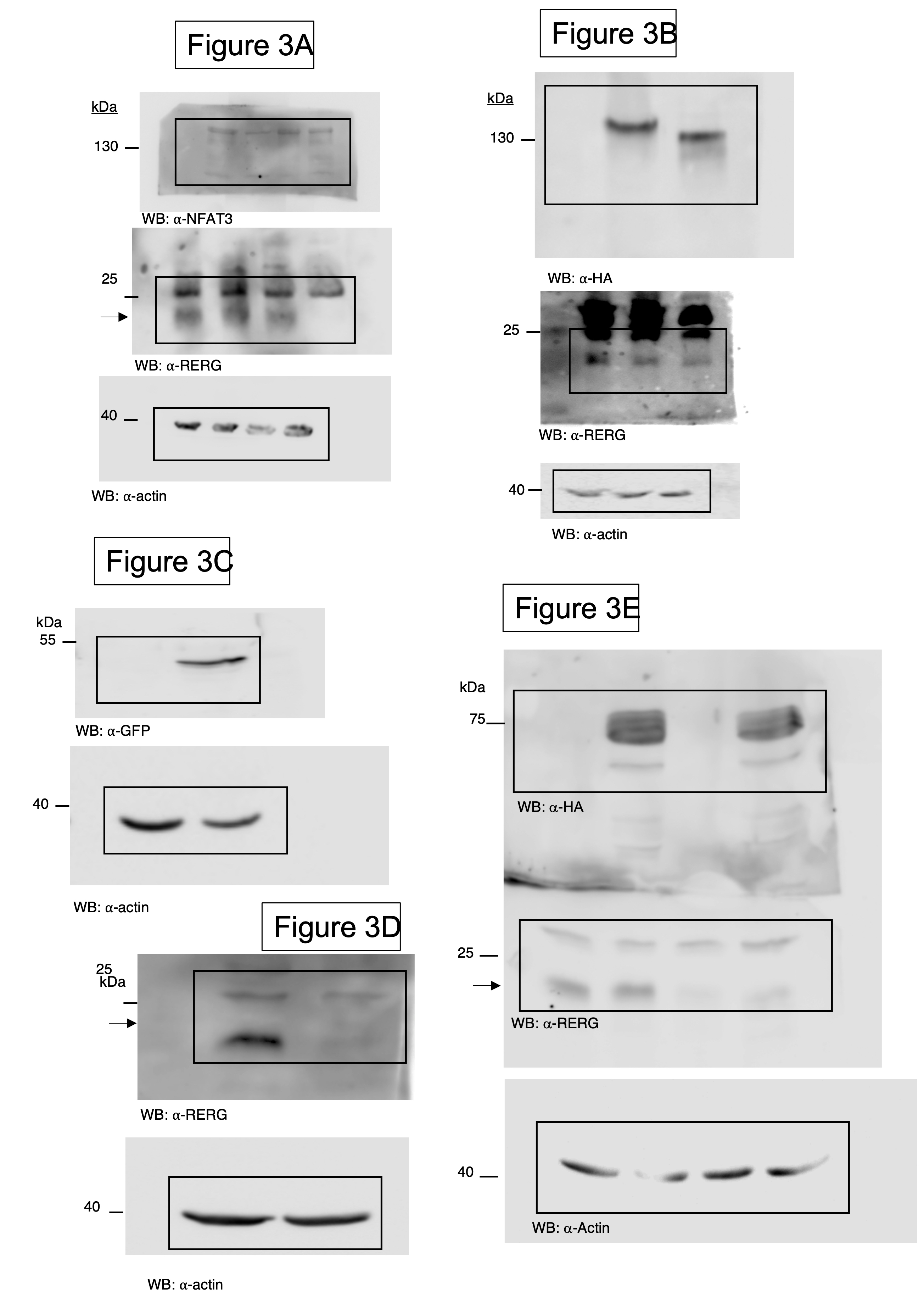

Supplement: Supplementary file 2 [file Image_2.jpeg]
